# *ZmPRN1* Negatively Regulates Salt Stress Tolerance by Modulating ROS Homeostasis in Maize (*Zea mays* L.)

**DOI:** 10.3390/plants15101585

**Published:** 2026-05-21

**Authors:** Lei Ma, Wenzong Li, Ke Zhang, Qingyun Zhang, Hua Xu, Baobao Wang, Lei Wang, Junjie Zou

**Affiliations:** 1 Biotechnology Research Institute, Chinese Academy of Agricultural Sciences, Beijing 100081, China; malei20520@163.com (L.M.); lwzm1010@163.com (W.L.); zhangk4840@163.com (K.Z.); 15756291121@163.com (H.X.); wangbaobao@caas.cn (B.W.); 2 National Nanfan Research Institute (Sanya), Chinese Academy of Agricultural Sciences, Sanya 572025, China; sdwfzqy1996@163.com; 3 Yazhouwan National Laboratory, Sanya 572024, China; 4 The Shennong Seed Industry Laboratory, Zhengzhou 450002, China

**Keywords:** chloroplast, maize, reactive oxygen species, salt stress

## Abstract

Soil salinization is a major abiotic stress limiting maize (*Zea mays* L.) growth and productivity worldwide. Recently, many genes involved in salt stress have been identified. However, the molecular mechanisms underlying salt tolerance in maize remain largely elusive. In this study, we identified a member of the ZmPIRIN family genes, *ZmPRN1*, acting as a negative regulator in response to salt stress. The expression levels of *ZmPRN1* were down-regulated under salt and H_2_O_2_ treatment. Subcellular localization analysis showed that ZmPRN1 is localized to the chloroplast. Under salt stress, the *Zmprn1*-Mu mutant exhibited higher survival rates and lower reactive oxygen species (ROS) accumulation compared to wild-type plants. Whereas, *ZmPRN1* overexpression lines were more sensitive to salt stress, and had higher ROS levels and lower chlorophyll content than wild-type plants. Transcriptome analysis showed that the differentially expressed genes (DEGs) were mainly involved in the oxidation-reduction process. Furthermore, yeast-two hybrid and split-luciferase complementation assays revealed that ZmPRN1 can interact with the chloroplast NDH complex subunit NDF4, the RING-type E3 ubiquitin ligase RING371, and the auxin-responsive protein IAA27. Collectively, our findings demonstrated that *ZmPRN1* negatively regulates salt tolerance in maize by modulating ROS homeostasis, providing a valuable genetic resource for breeding salt-tolerant maize varieties.

## 1. Introduction

Maize (*Zea mays* L.) is one of the most important cereal crops worldwide, playing a critical role in food production, livestock development, and industrial processing [1,2]. However, soil salinization has become a major abiotic constraint limiting maize production, affecting more than 20% of cultivated land globally, and this trend is continuously worsening [3,4,5,6]. Salt stress triggers ionic imbalance, osmotic stress, and oxidative damage, which severely inhibit root development, photosynthesis, and biomass accumulation in maize, ultimately leading to yield reduction or even complete crop failure [7,8,9,10,11]. As a typical glycophyte, maize is highly sensitive to salt stress, and improving its salt tolerance is of great practical significance for the development of saline-alkali lands and global food security [12,13]. Therefore, elucidating the molecular regulatory network underlying salt stress responses in maize and identifying key salt tolerance genes will provide important theoretical foundations and genetic resources for breeding salt-tolerant maize varieties.

Plants have evolved complex mechanisms to cope with salt stress, including osmotic regulation, ion homeostasis maintenance, and oxidative stress damage repair [14,15,16,17,18]. Under salt stress conditions, the level of reactive oxygen species (ROS) increases significantly in plant cells [7,8,19,20]. ROS mainly include hydrogen peroxide (H_2_O_2_), superoxide anion (O_2_^•−^), hydroxyl radical (HO^•^), and singlet oxygen (^1^O_2_), and are primarily produced in chloroplasts, mitochondria, peroxisomes, and the apoplast [21]. ROS have a dual function: at low concentrations, they act as important signaling molecules involved in various biological processes such as cell proliferation, differentiation, and stress responses; however, excessive accumulation of ROS leads to oxidative damage to DNA, proteins, and membrane lipids, and ultimately induces cell death [22,23,24]. Therefore, maintaining ROS homeostasis is essential for normal plant growth, development, and stress adaptation. Plants have evolved both enzymatic and non-enzymatic ROS scavenging systems to maintain ROS balance. The enzymatic system mainly includes superoxide dismutase (SOD), catalase (CAT), and ascorbate peroxidase (APX), while the non-enzymatic system includes antioxidants such as glutathione, ascorbate, and carotenoids [23,25,26]. Salt stress induces the expression of ROS scavenging-related genes, thereby reducing ROS accumulation, alleviating oxidative damage, and enhancing plant salt tolerance [27].

In maize, salt stress-induced ROS elevation represses the expression of *ZmmiR169q*, which relieves the transcriptional inhibition of its target gene *ZmNF-YA8*, subsequently activating the expression of the peroxidase gene *ZmPER1* and significantly enhancing the ROS scavenging capacity of the antioxidant system, thereby improving salt tolerance [28]. Similarly, salt-induced ROS repress the accumulation of miR408, promoting the transcription of its target genes *ZmLAC9* and *ZmLAC18*, which in turn regulate the polymerization of lignin monomers and strengthen cell wall structure to withstand salt stress damage [29]. Additionally, the ABA-stress-ripening gene *ZmASR6* was recently identified as a positive regulator of salt tolerance, and its knockout leads to increased ROS accumulation and reduced stress tolerance in maize [30]. In rice (*Oryza sativa* L.), the *OsCYBDOMG* gene, encoding a cytochrome b561 domain-containing protein, enhances salt tolerance by increasing ascorbate (AsA) content and its redox status; a natural variation from glycine (G) to aspartic acid (D) at position 57 of its DM13 domain significantly improves its catalytic activity, conferring a salt-tolerant phenotype [31]. Overexpressing *OsSTRK1* significantly enhances rice salt tolerance by activating catalase activity and reducing H_2_O_2_ accumulation [32]. In soybean (*Glycine max* L.), B-type heat shock factor HSFB2b enhances salt tolerance by activating the expression of flavonoid biosynthesis-related genes and repressing the expression of *GmNAC2*, a negative regulator of flavonoid synthesis, thereby increasing flavonoid accumulation [33]. In maize, the flavanone 3-hydroxylase gene *ZmF3H6* was recently shown to positively regulate salt tolerance by participating in flavonoid biosynthesis [34]. Similarly, the transcription factor ZmWRKY82 was recently shown to directly activate *ZmCHI6* expression, promoting flavonoid accumulation and ROS scavenging under saline-alkali stress, further supporting the importance of flavonoid-mediated antioxidant defense in maize [35]. Furthermore, the major alkali tolerance gene *AT1/GS3* has been shown to promote the efflux of stress-induced ROS by regulating the phosphorylation status of aquaporins, thereby alleviating oxidative stress damage and improving crop salt-alkali tolerance, and knockout of *AT1* via gene editing significantly enhances salt-alkali tolerance in monocot crops such as sorghum, foxtail millet, rice, and maize [36]. Collectively, these studies indicate that plants have evolved multilayered regulatory networks for ROS homeostasis, involving transcription factors, microRNAs, antioxidant enzymes, and transmembrane transporters. However, the key factors and molecular mechanisms regulating ROS homeostasis in maize remain to be further elucidated.

Pirin (PRN) is a highly conserved protein among mammals, plants, fungi, and even prokaryotic organisms [37,38]. In plants, pirin proteins are encoded by small gene families and feature a highly conserved N-terminal region containing two cupin domains and a metal ion-binding site composed of three histidine residues and one glutamic acid residue [37,38]. The first plant pirin gene identified was from tomato (*Solanum lycopersicum* L.), and its expression was shown to be closely associated with cell death [39]. The *Arabidopsis thaliana* genome encodes four pirin members. Among them, AtPRN1 interacts with the G protein α subunit GPA1 and is involved in ABA- and light-mediated seed germination and early seedling growth [40]. AtPRN1 also exhibits quercetinase activity and participates in the regulation of quercetin levels; notably, *prn1* mutants accumulate higher levels of quercetin and display enhanced tolerance to UV-C stress, surviving under UV-C irradiation conditions that are lethal to wild-type plants, indicating that *AtPRN1* negatively regulates abiotic stress tolerance [41]. *AtPRN2* is involved in pathogen response and lignin accumulation [42,43]. In wheat (*Triticum aestivum* L.), the pirin gene family has also been characterized and shown to be involved in stress responses [44]. However, functional studies of pirin genes in crops other than *Arabidopsis* remain limited, and their roles and molecular mechanisms in response to abiotic stresses such as salt stress require further investigation.

In this study, we identified a maize pirin family gene, *ZmPRN1*, and found that it negatively regulates salt stress tolerance. Through Mu transposon insertion mutant analysis, the *Zmprn1*-Mu mutant exhibited enhanced salt tolerance and lower ROS accumulation under salt stress conditions. Whereas, *ZmPRN1* overexpression lines were sensitive to salt stress and had higher ROS accumulation and lower chlorophyll content than wild-type plants. ZmPRN1 proteins are localized to the chloroplast, suggesting that they may participate in salt stress responses through chloroplast redox pathways. Transcriptome analysis further supported the involvement of *ZmPRN1* in the regulation of oxidation-reduction processes. Moreover, we screened and validated the interacting proteins of ZmPRN1, including the chloroplast NDH complex subunit NDF4, the RING-type E3 ubiquitin ligase RING371, and the auxin-responsive protein IAA27, implying that *ZmPRN1* may regulate salt stress responses by affecting photosynthesis, ubiquitin-dependent degradation, and auxin signaling pathways.

## 2. Results

### 2.1. Expression Patterns and Subcellular Localization of ZmPRN1

Through genome-wide analysis and phylogenetic tree analysis, there were six pirin-encoding genes were identified in the maize genome. ZmPRN1 was most closely related to ZmPRN2, while ZmPRN5 and ZmPRN6 formed a closely related cluster, and the remaining two members, ZmPRN3 and ZmPRN4, were more distantly related to the other family members (Figure S1). The N-terminal domains of these six ZmPRNs contain highly conserved metal ion binding sites composed of three histidine (His) residues and one glutamic acid (Glu) (Figure S2). Analysis of the expression levels of *ZmPRNs* in various tissues showed that these *ZmPRNs* have different expression patterns (Figure S3). *ZmPRN1* has higher expression levels in leaves, *ZmPRN5* transcripts can only be detected in the endosperm at 12 days after pollination, whereas there are no transcripts of *ZmPRN6* detected in maize tissues (Figure S3). Analyses of the cis-acting elements in the promoter region of *ZmPRN1* using PlantCARE database (https://bioinformatics.psb.ugent.be/webtools/plantcare/html/, accessed on 15 May 2021) revealed that there are multiple cis-acting elements associated with phytohormone responses and stress responses, suggesting that *ZmPRN1* may be involved in the integration of hormone and stress signaling pathways (Table S1).

**Figure 1:**
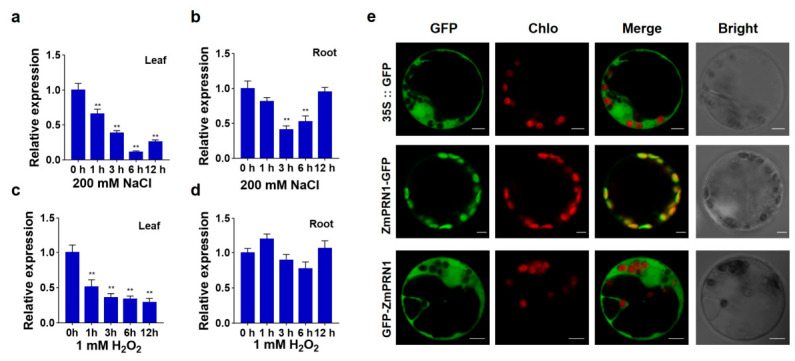
Expression patterns and subcellular localization of ZmPRN1. (**a**,**b**) Expression levels of *ZmPRN1* in leaves (**a**) and roots (**b**) treated with 200 mM NaCl at indicated time. Two-week-old maize seedlings were sampled at 0, 1, 3, 6, and 12 h after treatment, and the relative expression levels of *ZmPRN1* were determined by RT-qPCR. (**c**,**d**) Expression levels of *ZmPRN1* in leaves (**c**) and roots (**d**) treated with 1 mM H_2_O_2_ at the indicated time. Two-week-old maize seedlings were sampled at 0, 1, 3, 6, and 12 h after treatment, and the relative expression levels of ZmPRN1 were determined by RT-qPCR. *ZmTUB5* was used as an internal control. Data are presented as mean ± SD (*n* = 3). *p* values were determined by two-sided *t*-test. ** *p* < 0.01. (**e**) Subcellular localization of ZmPRN1. The full-length of ZmPRN1 CDS was fused to GFP at the N-terminus (*35S::ZmPRN1-GFP*) or C-terminus (*35S::GFP-ZmPRN1*) driven by 35S promoter. Images were acquired using a Zeiss LSM 980 confocal microscope. Green fluorescence represents GFP signal (excitation wavelength 488 nm), and red fluorescence represents chloroplast autofluorescence (excitation wavelength 561 nm). The yellow color in the merged images indicates co-localization of GFP (green) and chloroplast autofluorescence (red). Emission was collected at 500–550 nm for GFP and 650–750 nm for chloroplast autofluorescence. Scale bars = 5 μm in (**e**).

RT-qPCR was conducted to analyze the transcription changes of *ZmPRN1* after treatment with 200 mM NaCl or 1 mM H_2_O_2_. Under 200 mM NaCl treatment, *ZmPRN1* expression levels were downregulated in both leaves and roots, reaching its lowest level at 6 h and 3 h post-treatment, respectively (Figure 1a,b). Under 1 mM H_2_O_2_ treatment, *ZmPRN1* expression levels were similarly downregulated in leaves (Figure 1c), whereas no significant change was observed in roots (Figure 1d). These expression patterns suggested that *ZmPRN1* responds transcriptionally to both salt and oxidative stresses in a tissue-specific manner.

The subcellular localization of ZmPRN1 protein was initially predicted using WoLFPSORT (https://wolfpsort.hgc.jp/ (accessed on 15 May 2021)) and found that ZmPRN1 protein has chloroplast localization. To confirm this, transient expression of *35S::ZmPRN1-GFP* or *35S::GFP-ZmPRN1* fusion transgene in maize protoplasts was conducted. The results showed that the GFP green fluorescence signals match the red chlorophyll autofluorescence signals in *35S::ZmPRN1-GFP* transformed protoplasts. Whereas in the *35S::GFP-ZmPRN1* transformed protoplasts, the GFP green fluorescence was present in the cytoplasm, and no GFP fluorescence signals were detected in the chloroplast (Figure 1e). Taken together, these findings indicate that ZmPRN1 is localized in the chloroplasts and may play important roles in response to oxidative stresses.

### 2.2. Loss-of-Function of ZmPRN1 Enhanced Plants’ Tolerance to Salt Stress

**Figure 2:**
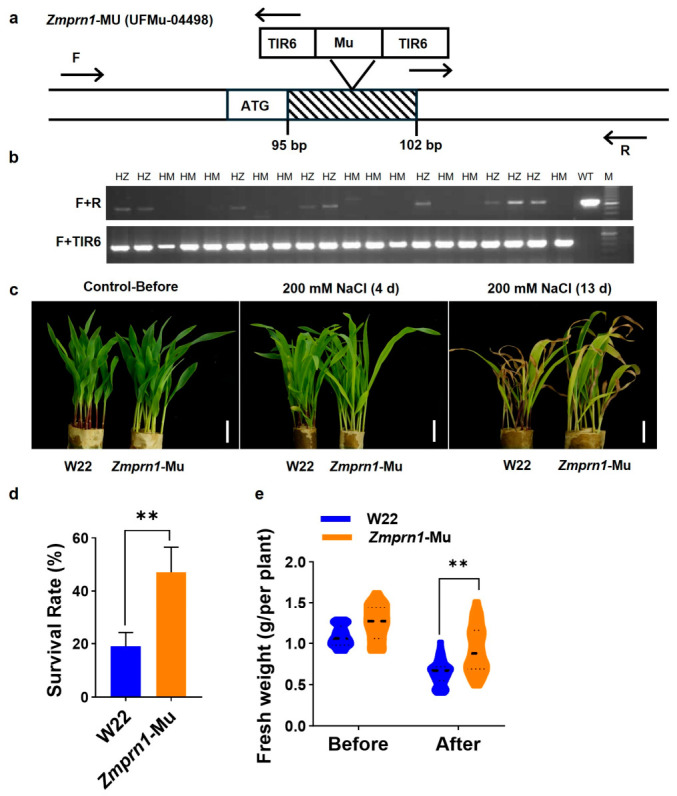
*ZmPRN1* negatively regulates salt tolerance in maize. (**a**) Schematic diagram of the Mu transposon insertion site in the *ZmPRN1* gene (UFMu-04498). The insertion position of the Mu transposon, the primer TIR6 used for Mu element detection, and the primers F and R used for genomic amplification to determine the genotype are indicated. (**b**) PCR-based genotyping of the *ZmPRN1* Mu insertion mutants. M: 100 bp DNA Marker; WT: wild-type plants; HM: homozygous mutants; HZ: heterozygous mutant. (**c**–**e**) Phenotypic comparison of W22 wild-type and *Zmprn1*-Mu mutant under salt stress. Representative images of 12-day-old seedlings treated with 200 mM NaCl (**c**). Survival rate (**d**). Fresh weight (**e**). Scale bars = 3 cm in (**c**). Data in (**d**,**e**) are presented as mean ± SD from three biological replicates (*n* = 11 plants per replicate). *p* values were determined by two-sided *t*-test. ** *p* < 0.01.

To investigate the role of *ZmPRN1* in response to salt stress in maize, we obtained a Mu transposon insertion mutant, UFMu-04498, from the Uniform-Mu mutant resource center. The Uniform-Mu population, generated by introgressing an active Robertson’s Mutator (Mu) transposon into the W22 inbred line, represents a widely used functional genomics resource that enables systematic reverse genetics studies in maize [45]. Genotyping was performed using specific primers designed based on the *ZmPRN1* genomic sequence and the Mu transposon insertion site, leading to the identification of a homozygous Mu insertion mutant. After four successive generations of self-pollination and genotypic selection, we obtained a stable homozygous Mu insertion mutant line, which was designated as *Zmprn1*-Mu for subsequent experiments (Figure 2a,b).

To evaluate the role of *ZmPRN1* in response to salt stress, *Zmprn1*-Mu mutant and corresponding wild-type W22 plants were subjected to 200 mM NaCl treatment under controlled liquid growth conditions. After 4 days of NaCl treatment, chlorosis was observed at the tips of the first leaves in wild-type plants, whereas no obvious phenotypic changes were detected in the *Zmprn1*-Mu plants, suggesting that the mutant exhibited initial tolerance to salt stress. After 13 days of NaCl treatment, *Zmprn1*-Mu mutant exhibited significantly more tolerance to salt stress compared to the wild-type plants (Figure 2c). Consistently, statistical analysis revealed that both the survival rate and fresh weight of the mutant plants were significantly higher than those of the wild-type plants (Figure 2d,e). These results indicated that *ZmPRN1* functions as a negative regulator of salt tolerance in maize, as its loss of function leads to enhanced resistance to salt stress.

**Figure 3:**
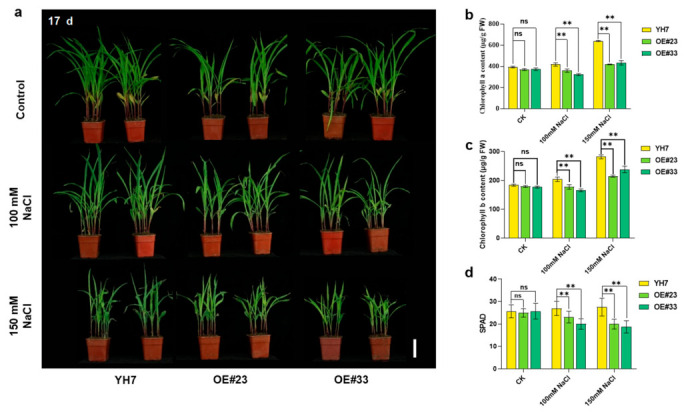
Phenotype of ZmPRN1 overexpression lines under salt stress. (**a**) Salt stress phenotype of ZmPRN1 overexpression lines compared with wild-type plants under normal and salt stress conditions in soil. (**b**) The concentrations of chlorophyll *a* in wild-type and ZmPRN1 overexpression plants treated with 100 mM or 150 mM NaCl. (**c**) The concentrations of chlorophyll *b* in wild-type and ZmPRN1 overexpression plants treated with 100 mM or 150 mM NaCl. (**d**) SPAD value of leaves of wild type and *ZmPRN1* overexpression plants under normal and salt stress conditions. Scale bar = 8 cm in (**a**). Data in (**b**–**d**) are presented as mean ± SD from three biological replicates. *p* values were determined by two-sided *t*-test. ** *p* < 0.01; ns = not significant.

To further confirm the negative role of *ZmPRN1* in salt stress, the *ZmPRN1* overexpression lines were obtained and used for salt treatment. RT-qPCR analysis showed that the expression levels of *ZmPRN1* overexpression lines were significantly higher than those of wild-type plants (Figure S4a). Then, two overexpression lines (OE#23, OE#33) were selected for salt treatment in soil. After 10 days of growth in soil, there was no significant difference between wild-type plants (YH7) and overexpression lines under normal growth conditions. 100 mM or 150 mM NaCl treatment inhibited plant growth, and the plant height of overexpression lines was shorter than that of the wild-type plants. And the leaf colour of overexpression lines was lighter than that of wild-type plants (Figure S4b). After treatment with 100 mM or 150 mM NaCl for 17 d, the difference between wild-type plants and *ZmPRN1* OE lines was distinct (Figure 3a). The chlorophyll *a* and chlorophyll *b* contents in the leaves of *ZmPRN1* OE lines were significantly lower than those in the leaves of wild-type plants (Figure 3b,c). The chlorophyll content was also measured using a SPAD meter (SPAD-502 Plus, Konica Minolta, Japan). And the results showed that salt stress significantly causes a decrease in the total chlorophyll content in the *ZmPRN1* OE lines compared to the wild type plants (Figure 3d). These results indicated that overexpressing *ZmPRN1* in plants causes increased sensitivity to salt stress and chlorophyll function may be impaired severely in the *ZmPRN1* OE lines compared to the wild-type plants.

Subsequently, we performed transcriptome sequencing analysis on leaves of *Zmprn1*-Mu mutant and wild-type plants following 48 h of 200 mM NaCl treatment. A total of 859 upregulated genes and 376 downregulated genes were identified as differentially expressed genes (DEGs) between the mutant and wild-type under salt stress conditions (Figure S5a). To explore the potential functions of these DEGs, Gene Ontology (GO) enrichment analysis was conducted. And the results showed that the DEGs were significantly enriched in the “oxidation-reduction process” category (Figure S5b). Furthermore, we examined the expression patterns of genes involved in enzymatic and non-enzymatic ROS scavenging systems. Heatmap analysis revealed that many ROS scavenging-related genes, including those encoding peroxidases (PODs), peroxiredoxins (PrxRs), CATs, and flavonoid synthases (FLSs), were significantly upregulated in the *Zmprn1*-Mu mutant compared with wild-type plants under salt stress (Figure S5c). These results indicated that Loss-of-function of *ZmPRN1* may be involved in regulating redox homeostasis under salt stress.

### 2.3. ZmPRN1 Regulates Reactive Oxygen Species Homeostasis Under Salt Stress

**Figure 4:**
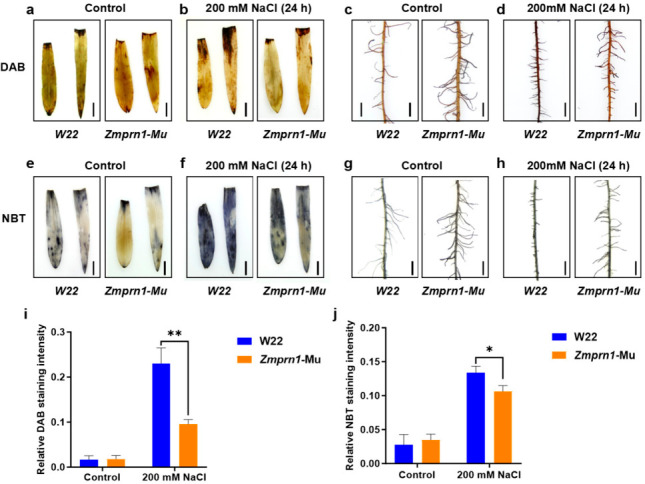
Mutation of *ZmPRN1* leads to reduced ROS accumulation under NaCl treatment. (**a**–**d**) DAB staining of leaves (**a**,**b**) or roots (**c**,**d**) of wild-type and *Zmprn1*-Mu plants treated with 200 mM NaCl for 24 h. (**e**–**h**) NBT staining of leaves (**e**,**f**) or roots (**g**,**h**) of wild-type and *Zmprn1*-Mu plants treated with 200 mM NaCl for 24 h. Scale bars = 3 cm in (**a**–**h**). (**i**,**j**) Quantitative analysis of relative DAB (**i**) and NBT (**j**) staining intensity. The integrated density of stained areas was measured using ImageJ software (version 1.54f) and normalized to total leaf area. *p* values were determined by two-sided *t*-test. * *p* < 0.05, ** *p* < 0.01.

**Figure 5:**
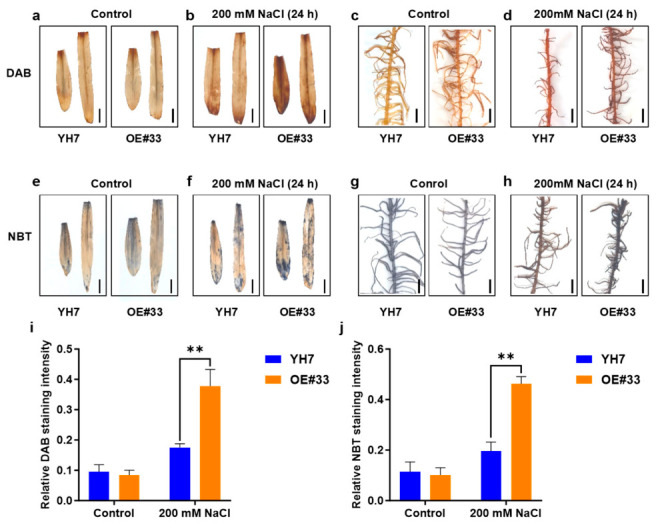
Overexpression of *ZmPRN1* leads to increased ROS accumulation under NaCl treatment. (**a**–**d**) DAB staining of leaves (**a**,**b**) or roots (**c**,**d**) of wild-type (YH7) and *ZmPRN1*-overexpressing (OE#33) plants treated with 200 mM NaCl for 24 h. (**e**–**h**) NBT staining of leaves (**e**,**f**) or roots (**g**,**h**) of wild-type(YH7) and *ZmPRN1*-overexpressing plants (OE#33) treated with 200 mM NaCl for 24 h. Scale bars = 2 cm in (**a**–**h**). (**i**,**j**) Quantitative analysis of relative DAB (**i**) and NBT (**j**) staining intensity. The integrated density of stained areas was measured using ImageJ software (version 1.54f) and normalized to total leaf area. *p* values were determined by two-sided *t*-test. ** *p* < 0.01.

Given that the transcriptome analysis suggested a potential role for *ZmPRN1* in redox regulation, we further examined the accumulation of ROS in plants under salt stress. ROS, including H_2_O_2_ and O_2_^•−^, are key signaling molecules that can cause oxidative damage when accumulated to excessive levels under stress conditions [7,8,19,20,21]. DAB (3,3-diaminobenzidine) staining and NBT (nitro blue tetrazolium) staining were performed to detect the levels of H_2_O_2_ and O_2_^•−^, respectively. After treatment with 200 mM NaCl for 24 h, the ROS levels in the leaves and roots of *Zmprn1*-Mu mutant plants, *ZmPRN1*-overexpressing plants, and their corresponding wild-type controls were analyzed (Figure 4 and Figure 5). Under normal growth conditions, minimal staining was observed in the leaves and roots of W22 and *Zmprn1*-Mu plants, indicating low basal levels of ROS. Under salt stress, strong DAB and NBT staining intensities were detected in both leaves and roots of wild-type plants (W22), indicating substantial accumulation of H_2_O_2_ and O_2_^•−^. In contrast, the *Zmprn1*-Mu mutant exhibited markedly weaker staining intensities under the same treatment conditions, with ROS accumulation levels significantly lower than those observed in wild-type plants (Figure 4). As for *ZmPRN1*-overexpressing plants (OE#33) and their corresponding wild-type plants (YH7), there were no significant differences in DAB and NBT staining intensities between YH7 and OE#33 plants under normal growth conditions. Under 200 mM NaCl treatment for 24 h, *ZmPRN1*-overexpressing plants exhibited significantly stronger DAB and NBT staining intensities compared to YH7, indicating elevated ROS levels in OE#33 relative to YH7 under salt stress (Figure 5). These results revealed that loss of *ZmPRN1* function reduces salt stress-induced ROS accumulation, whereas overexpression of *ZmPRN1* conferred enhanced ROS accumulation under salt stress, suggesting that *ZmPRN1* negatively regulates ROS homeostasis under salt stress.

### 2.4. Screening and Validation of ZmPRN1-Interacting Proteins

To further elucidate the function of *ZmPRN1* in maize, we performed yeast two-hybrid screening to identify its interacting proteins. Given that the full-length ZmPRN1 exhibited autoactivation activity (Figure S6), which would interfere with the screening, we divided it into four segments (A, B, C, and D) based on its conserved functional domains. The longest segment lacking autoactivation activity (the BCD segment) was selected as bait for yeast library screening using the mating method, which allows for efficient large-scale screening of protein interactions. After multiple rounds of screening on selective media, positive clones were obtained, and the inserted fragments from the pGADT7 vectors were extracted and subjected to sequencing analysis. Candidate genes associated with stress responses, hormone responses, and chloroplast functions were selected for subsequent validation based on their potential relevance to *ZmPRN1* function (Table S2).

**Figure 6:**
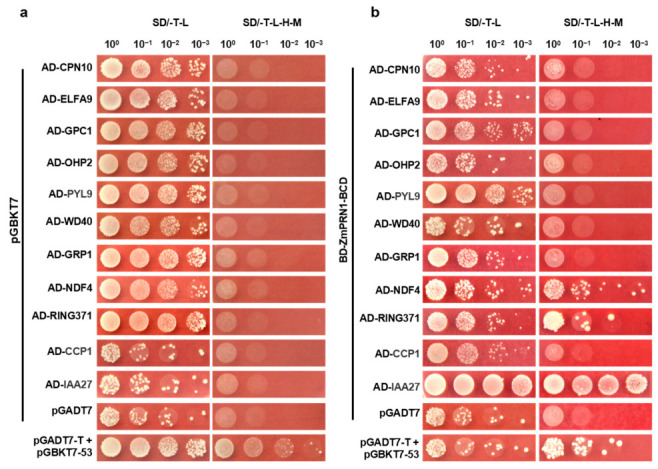
Yeast two-hybrid validation of the interaction between ZmPRN1 and candidate proteins. (**a**) Detection of autoactivation activity of candidate proteins. Each candidate protein fused with the GAL4 activation domain (AD) was co-transformed with the pGBKT7 empty vector into the yeast strain AH109. (**b**) Validation of interactions between ZmPRN1 and candidate proteins. Co-transformation of pGBKT7-53 with pGADT7-T served as a positive control.

To confirm the autoactivation status of the candidate proteins, we cloned each candidate gene into the pGADT7 vector and co-transformed it with the pGBKT7 empty vector into yeast competent cells. The results showed that none of the candidate proteins exhibited autoactivation activity (Figure 6a), confirming their suitability for interaction assays. Subsequently, we co-transformed the candidate genes with pGBKT7-ZmPRN1-BCD into yeast competent cells. We found that ZmPRN1 interacted with the NDH subunit NDF4 (Zm00001d021515), the RING-type E3 ubiquitin ligase RING371 (Zm00001d034998), and the auxin-responsive protein IAA27 (Zm00001d037774), as evidenced by the growth of yeast colonies on selective media (Figure 6b).

**Figure 7:**
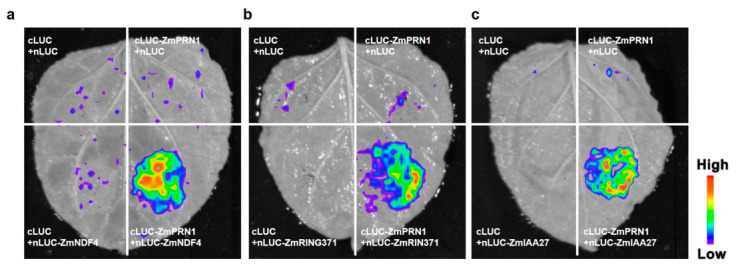
Validation of interactions between ZmPRN1 and candidate proteins by split-luciferase complementation assay. (**a**) The interaction between ZmPRN1 and ZmNDF4 as tested by split-luciferase complementation in *Nicotiana benthamiana.* (**b**) The interaction between ZmPRN1 and ZmRING371 as tested by split-luciferase complementation in *Nicotiana benthamiana.* (**c**) The interaction between ZmPRN1 and ZmIAA27 as tested by split-luciferase complementation in *Nicotiana benthamiana*.

To confirm these interactions in a plant system, we performed dual-luciferase complementation assays in leaves of *Nicotiana benthamiana*. The results further validated these protein–protein interactions, with significant luciferase activity detected when the interacting pairs were co-expressed (Figure 7). NDF4 is a subunit of the chloroplast NDH complex, which is involved in cyclic electron transport around photosystem I, photosynthesis, and stress responses [46,47,48,49,50]. RING371, functioning as an E3 ubiquitin ligase, plays an important role in the ubiquitin-proteasome pathway and abiotic stress responses by targeting specific substrates for degradation [51,52,53,54,55,56]. IAA27 is a key auxin-responsive protein belonging to the Aux/IAA family, which functions as a transcriptional repressor through interaction with ARF transcription factors [57,58]. Taken together, these results indicated that ZmPRN1 may participate in the regulatory networks governing photosynthesis, stress responses, and hormone signaling through protein–protein interactions, providing new insights into the molecular mechanisms by which ZmPRN1 modulates salt tolerance in maize.

## 3. Discussion

In this study, we identified a maize pirin family gene, *ZmPRN1*, and demonstrated its role as a negative regulator of salt tolerance. Loss-of-function *Zmprn1*-Mu mutants exhibited significantly enhanced salt tolerance compared with wild-type plants, as evidenced by higher survival rates, increased fresh weight, and reduced ROS accumulation under salt stress conditions. *ZmPRN1* overexpression lines were sensitive to salt stress, with decreased plant height, and had higher ROS levels and lower chlorophyll content compared to wild-type under salt stress. These results establish *ZmPRN1* as a key negative modulator of salt stress responses in maize.

Transcriptome analysis revealed that genes differentially expressed in *Zmprn1*-Mu mutants were significantly enriched in the “oxidation-reduction process” category, with a subset of enzymatic and non-enzymatic antioxidant genes being upregulated. DAB and NBT staining showed that *Zmprn1*-Mu mutants accumulated lower levels of ROS under salt stress compared with wild-type plants. These results indicate that ZmPRN1 negatively regulates ROS homeostasis under salt stress. The subcellular localization of ZmPRN1 to the chloroplast further supports its role in redox regulation, as chloroplasts are a major site of ROS production under stress conditions. In *Arabidopsis*, AtPRN1 exhibits quercetinase activity and negatively regulates UV-C stress tolerance [41]. Given the structural conservation between ZmPRN1 and AtPRN1, it is plausible that ZmPRN1 may also possess quercetinase activity, thereby modulating ROS levels by degrading the antioxidant quercetin. Therefore, reduced ROS accumulation in the *Zmprn1*-Mu mutant may result from increased flavonoid antioxidant levels. However, direct evidence for quercetinase activity of ZmPRN1 and its contribution to salt tolerance requires further investigation.

Through yeast-two hybrid and split-luciferase complementation assays, we identified three ZmPRN1-interacting proteins: NDF4, RING371, and IAA27. Although their precise functions in salt stress responses remain to be validated, their known functions provide clues for understanding how *ZmPRN1* modulates salt tolerance. The chloroplast NADH dehydrogenase-like (NDH) complex mediates cyclic electron transport around photosystem I and maintains ATP/NADPH balance under stress conditions [46,47,48,49,50]. Overexpression of NDH complex subunits (e.g., *ZmNdhl1*, *ZmNdhl2*, and *OsNdhl*) enhances salt tolerance by increasing cyclic electron transport activity [59]. Given that ZmPRN1 localizes to the chloroplast, it may influence photosynthetic performance by interacting with NDH complex subunits. RING-type E3 ubiquitin ligases mediate ubiquitination and degradation of specific substrates in abiotic stress responses [51,52,53,54,55,56]. Studies in rice have identified such ligases involved in salt (e.g., OsRFI2), drought (e.g., OsRINGzf1), and heat (e.g., TT3.1) stress responses [60,61,62]. Rice OsPIRIN was found to interact with an E3 ligase named Salt-, ABA-, and Drought-Induced RING Finger Protein 1 (OsSADR1). OsSADR1 interacts with OsPIRIN and leads to OsPIRIN degradation [63]. By analogy, it is possible that ZmRING371 has a similar function, although ubiquitination assays were not performed in this study. Aux/IAA proteins function as transcriptional repressors in auxin signaling [57,58]. Aux/IAA genes play crucial roles in abiotic stress responses, with various members shown to enhance salt or drought tolerance through regulating ABA signaling, ROS scavenging, and antioxidant activities (e.g., *OsIAA18*, *IAA5/6/19*, *TrIAA27*) [64,65,66]. The interaction between ZmPRN1 and IAA27 suggests a potential link to auxin signaling, although direct evidence of auxin involvement in this process is not provided in the current study. Additionally, the effect of NDF4 mutation on ROS accumulation under salt stress remains to be demonstrated. Conducting validation experiments on these genes in response to salt stress will further elucidate and refine the findings pertaining to the ZmPRN1-mediated salt stress pathway. Collectively, the interactions between ZmPRN1 and these proteins suggest that *ZmPRN1* may be involved in photosynthesis, ubiquitin-dependent degradation, and auxin signaling, although the functional relevance of these interactions to salt tolerance requires further investigation.

Subcellular localization prediction of ZmPRN1 suggests that ZmPRN1 has a transit peptide and may have chloroplast localization. The subcellular localization assay showed that ZmPRN1 localizes to the chloroplast based on transient expression of the C-terminal GFP fusion protein (ZmPRN1-GFP) in maize protoplasts. The N-terminal GFP fusion (GFP-ZmPRN1) localized to the cytoplasm, likely because the N-terminally fused GFP tag physically obstructs the recognition and cleavage of the N-terminal chloroplast transit peptide, indicating that the N-terminal of ZmPRN1 is very important for its subcellular localization. Future validation using chloroplast fractionation coupled with immunoblot analysis would help to more reliably confirm the chloroplast localization of ZmPRN1. Nevertheless, the localization results from the C-terminal fusion, together with the bioinformatic prediction of a transit peptide, support that ZmPRN1 likely localizes to the chloroplast and is involved in regulating chloroplast redox homeostasis.

Our findings provide several novel insights into the regulation of salt tolerance in maize and plants. It represents the first characterization of a pirin family gene in maize salt stress responses, filling a gap in our understanding of pirin proteins beyond *Arabidopsis*. Notably, ZmPRN1 localizes to the chloroplast, distinct from the nuclear-localized AtPRN1, suggesting functional divergence of pirin proteins in plants. In addition, we identified three ZmPRN1-interacting proteins, NDF4, RING371, and IAA27, providing new perspectives on the multifaceted regulatory networks involving pirin proteins. Furthermore, this study expands the functional repertoire of the pirin family from cell death, seed germination, and pathogen responses to salt stress adaptation. From an application perspective, *ZmPRN1* represents a promising genetic resource for salt-tolerant maize breeding, as its knockout significantly enhances salt tolerance, similar to the strategy of targeting negative regulators such as *AT1* for crop stress tolerance improvement [36].

The precise molecular mechanism by which ZmPRN1 regulates ROS homeostasis remains to be elucidated, and the functional relevance of the identified protein–protein interactions (NDF4, RING371, and IAA27) in salt stress responses requires further experimental validation. Nevertheless, our findings demonstrate that *ZmPRN1* serves as a negative regulator of salt tolerance in maize and establish a foundation for future mechanistic investigations.

## 4. Materials and Methods

### 4.1. Plant Materials and Growth Conditions

Mu transposon insertion mutant UFMu-04498 was obtained from the Uniform-Mu mutant resource center. Genotyping was performed using specific primers designed based on the *ZmPRN1* genomic sequence and the Mu transposon insertion site to identify a homozygous Mu insertion mutant. After four successive generations of self-pollination and genotypic selection, a stable homozygous Mu insertion mutant line was obtained and designated as *Zmprn1*-Mu. The wild-type control used in this study was the W22 inbred line. In addition, the maize inbred line B73 was used for gene expression analysis. Maize seeds were germinated and grown in paper rolls moistened with water. Plants were grown in a greenhouse under controlled conditions with a 16-h light/8-h dark photoperiod at 28°C and 30% humidity.

The *ZmPRN1* overexpression transgenic lines were generated in the YH7 genetic background and provided by Bomei Xing’ao Technology Co., Ltd. (Beijing, China). Homozygous transgenic plants were obtained through self-pollination and confirmed by PCR genotyping and RT-qPCR expression analysis. Maize seeds were germinated and grown in pots containing a 1:1 (*v*/*v*) mixture of vermiculite and nutrient soil. The nutrient soil (Pindstrup, Ryomgaard, Denmark) had a pH of 6.0–6.5 (measured in water at a 1:2.5 soil-to-water ratio), and a total nitrogen content of 1.2%. Plants were maintained in a greenhouse under controlled conditions: 28 °C with a 16-h light/8-h dark photoperiod and 30% relative humidity. All plants were cultivated in pots measuring 8 cm in height and 6 cm in diameter.

### 4.2. Salt Treatment

For salt stress phenotyping of *Zmprn1-Mu* mutant in liquid solution, fifteen seeds per genotype were sown in paper rolls moistened with water and placed in a greenhouse. Uniform seedlings at the two-leaf stage were selected and subjected to treatment by replacing the water with a 200 mM NaCl solution. The 200 mM NaCl concentration was selected based on preliminary experiments for its ability to induce non-lethal stress phenotypes. Photographs were taken at 4 and 13 days after treatment, and survival rates and fresh weights were recorded. To analyze the expression patterns of *ZmPRN1* in response to salt and oxidative stress, B73 seedlings were treated with 200 mM NaCl. Leaves and roots were sampled at 0, 1, 3, 6, and 12 h after treatment for RNA extraction and RT-qPCR analysis. Time points (0, 1, 3, 6, and 12 h) were chosen to cover early and late transcriptional responses of *ZmPRN1*. For ROS staining, leaves and roots of *Zmprn1*-Mu, *ZmPRN1*-overexpressing plants, and their corresponding wild-type plants were collected 24 h after salt treatment for DAB and NBT staining to detect H_2_O_2_ and O_2_^•−^ accumulation, respectively. For transcriptome analysis, leaves were collected 48 h after salt treatment for RNA extraction and sequencing.

For salt stress phenotyping of *ZmPRN1*-overexpressing plants in soil, nine seeds per pot were sown and irrigated with water (control), 100 mM, or 150 mM NaCl solution. Seedlings were photographed at 10 d and 17 d after treatment, and chlorophyll content and SPAD values were measured at 17 d after treatment. All experiments were performed with three biological replicates.

### 4.3. H_2_O_2_ Treatment

Maize lines used for H_2_O_2_ sensitivity assays were grown using the paper roll culture method as described above. Two-week-old B73 seedlings were treated with 1 mM H_2_O_2_. Leaves and roots were collected at 0, 1, 3, 6, and 12 h after treatment, immediately frozen in liquid nitrogen, and used for total RNA extraction. The expression levels of *ZmPRN1* were then analyzed by RT-qPCR.

### 4.4. Quantitative Real-Time PCR (RT-qPCR) Analysis

Total RNA was extracted from plant samples using RNA prep pure plant kit (Tiangen, Beijing, China). First-strand cDNAs were synthesized with the TIANGEN reverse transcription kit according to the manufacturer’s instructions. Quantitative real-time PCR was performed using SuperReal PreMix Plus (SYBR Green; Tiangen, Beijing, China) on a 7500 real-time PCR detection system (Applied Biosystems, Waltham, MA, USA). *ZmTUB5* was used as the internal reference gene for normalization, and relative expression levels were calculated using the 2^−ΔΔCt^ method. Primers were listed in Table S2.

### 4.5. Histochemical Staining of O_2_^•−^ and H_2_O_2_

Histochemical staining of O_2_^•−^ and H_2_O_2_ was performed using nitroblue tetrazolium (NBT) and 3,3′-diaminobenzidine (DAB), respectively, as previously described [67]. Intact leaves and root segments were infiltrated with 0.2% NBT (Sigma, St. Louis, MO, USA) in 50 mM sodium phosphate buffer (pH 7.5) or 1 mg/mL DAB (Sigma, St. Louis, MO, USA) in 10 mM sodium phosphate buffer (pH 6.5), followed by vacuum infiltration for 10 min and incubation in the dark at 37 °C until color development. After staining, leaves and roots were washed with boiling ethanol for 10 min and then transferred to fresh ethanol. Images were captured using a digital camera. Leaf images were analyzed using ImageJ. Images were converted to 8-bit grayscale, and the threshold was adjusted to select stained areas. The integrated density of stained regions was measured and normalized to total leaf area. Relative staining intensity is expressed as integrated density per unit leaf area.

### 4.6. RNA-Seq

For transcriptome analysis, wild-type W22 and *Zmprn1*-Mu mutant plants were subjected to either salt treatment or control treatment. Leaves were collected immediately prior to treatment (0 h) and after 48 h of treatment for RNA extraction and sequencing. RNA libraries were sequenced on the Illumina NovaSeq 6000 platform (San Diego, CA, USA), generating approximately 20 million 150 bp paired-end reads per sample. Raw reads were trimmed and aligned to the maize B73 reference genome (Zm-B73-REFERENCE-GRAMENE-4.0) using HISAT2 (version 2.2.1). Gene expression levels were quantified, and differential expression analysis was performed using DESeq2 (version 1.10.1). Genes with |log2 fold change| > 1 and an adjusted *p*-value < 0.005 were considered differentially expressed. Two biological replicates were used for each genotype under each treatment condition. Sequencing and data analysis were performed by a commercial service provider.

### 4.7. Subcellular Localization

The coding sequence (CDS) of *ZmPRN1* was cloned into the pAN580 vector to generate a *35S::ZmPRN1-GFP* and *35S::GFP-ZmPRN1* fusion construct, which were transiently expressed in maize protoplasts, respectively, as previously described [68,69]. After incubation overnight for 16 h at room temperature, the transfected protoplasts were observed using a Zeiss LSM 980 (Zeiss, Oberkochen, Germany) confocal microscope. GFP fluorescence was detected with excitation at 488 nm and emission collected at 500–550 nm, while chloroplast autofluorescence was detected with excitation at 488 nm or 561 nm and emission collected at 650–750 nm.

### 4.8. Analysis of Chlorophyll Content

Chlorophyll content was determined using a plant chlorophyll content assay kit (Solarbio, Beijing, China, BC0995). Leaf samples were collected from wild-type and *ZmPRN1*-overexpressing plants grown under normal conditions or treated with 100 mM or 150 mM NaCl for 17 d. The experimental procedure was performed according to the manufacturer’s instructions. Absorbance values were measured at 645 nm (chlorophyll *a* absorption peak) and 663 nm (chlorophyll *b* absorption peak), and the concentrations of chlorophyll *a* and chlorophyll *b* were calculated accordingly.

### 4.9. SPAD Value Measurement

Leaf chlorophyll index was measured using a SPAD-502Plus chlorophyll meter (Konica Minolta, Tokyo, Japan). Wild-type and ZmPRN1-overexpressing plants were grown under normal conditions or treated with 100 mM or 150 mM NaCl for 17 d. For each plant, three different positions on the second leaf were selected for measurement, and the average value was recorded as the SPAD value of the leaf.

### 4.10. Yeast Two-Hybrid Library Screening

The coding sequence of *ZmPRN1* was divided into segments based on conserved functional domains and cloned into the pGBKT7 vector. Each bait construct was co-transformed with an empty pGADT7 vector into AH109 yeast competent cells and plated on SD/-T-L medium (synthetic dropout medium lacking Trp and Leu). Co-transformed yeast cells were then serially diluted in 10-fold steps and spotted onto SD/-T-L medium and SD/-T-L-H-M medium (synthetic dropout medium lacking Trp, Leu, His, and Met) to test for autoactivation and toxicity. The longest fragment lacking autoactivation activity and exhibiting no toxicity was selected as bait. The bait plasmid was then transformed into yeast, and library screening was performed using the mating method, as previously described [70]. Transformants were plated on SD/-T-L-H-M medium and incubated at 30 °C for 4–5 days. Positive colonies were selected and subjected to a second round of screening to confirm specific interactions with the bait protein. The prey fragments were identified by PCR, sequenced, and analyzed by BLAST (https://blast.ncbi.nlm.nih.gov, BLASTN 2.15.0) against the maize genome database.

### 4.11. Yeast Two-Hybrid (Y2H) Assay

The coding sequences of candidate prey proteins identified from the library screening were cloned into the pGADT7 vector. Each prey construct was co-transformed with empty pGBKT7 or bait plasmid (pGBKT7-ZmPRN1-BCD) into AH109 yeast competent cells using the PEG/LiAc method, as previously described [71]. Transformed cells were plated on SD/-Leu/-Trp medium and incubated at 30°C for 3 days. Subsequently, co-transformed yeast cells were serially diluted in 10-fold steps and spotted onto SD/-L/-T medium (growth control) and SD/-L/-T/-H/-M medium (selective medium) to test for autoactivation and protein–protein interactions. The combination pGADT7-T + pGBKT7-53 was used as a positive control, and pGADT7 + pGBKT7 was used as a blank control.

### 4.12. Split-Luciferase Complementation Assay

The coding sequence of *ZmPRN1* was cloned into the pCAMBIA1300-cLUC vector, and the coding sequences of candidate proteins were cloned into the pCAMBIA1300-nLUC vector. The resulting constructs were transformed into *Agrobacterium tumefaciens* strain GV3101. Healthy and young *Nicotiana benthamiana* leaves were selected for agroinjection. After injection, plants were kept in darkness for 24 h and then transferred to a 16-h light/8-h dark photoperiod for 24–48 h. Luciferase activity was detected using a NightShade LB985 Plant Imaging System (Berthold Technologies, Bad Wildbad, Germany) after application of 20 mg/mL D-luciferin potassium salt (Gold Biotech, St. Louis, MO, USA), as previously described [72].

### 4.13. Statistical Analysis

In all graphs, data are means ± standard deviation (s.d.) of at least three biological replicates, or at least three biological replicates plus three technical replicates. Statistical analysis was performed using a two-tailed Student’s *t*-test.

## 5. Conclusions

In this study, we identified *ZmPRN1*, a member of the maize PIRIN gene family, as a negative regulator of salt tolerance. Loss-of-function of *ZmPRN1* enhanced salt tolerance in maize, while its overexpression increased salt sensitivity. Our results demonstrated that ZmPRN1 localizes to the chloroplast and negatively regulates ROS homeostasis under salt stress. Transcriptome analysis revealed that differentially expressed genes in the *Zmprn1*-Mu mutant were significantly enriched in oxidation-reduction processes. Furthermore, we identified three ZmPRN1-interacting proteins—NDF4, RING371, and IAA27—through yeast two-hybrid and split-luciferase complementation assays, suggesting potential links to photosynthesis, ubiquitin-dependent degradation, and auxin signaling. Collectively, our findings establish *ZmPRN1* as a key negative modulator of salt tolerance in maize and provide a valuable genetic resource for breeding salt-tolerant maize varieties. Future studies are needed to elucidate the functional relevance of the identified protein–protein interactions and to explore the potential of *ZmPRN1* as a target for genetic improvement.

## Data Availability

All data generated or analysed during this study are available within the article or upon request from the corresponding authors.

## Supplementary Materials

The following supporting information can be downloaded at: https://www.mdpi.com/article/10.3390/plants15101585/s1, Figure S1: Phylogenetic tree of ZmPRN1 and its homologous proteins; Figure S2: Amino acid sequence alignment of ZmPRNs; Figure S3: Expression atlas of six *ZmPRNs* in maize; Figure S4: Phenotype of *ZmPRN1* overexpression lines treated with NaCl for 10 days; Figure S5: Transcriptomic profiling of wild-type and *Zmprn1*-Mu seedlings under salt stress; Figure S6: Functional domains and autoactivation activity of ZmPRN1; Table S1: Cis-acting elements in the promoter region of *ZmPRN1*; Table S2: Information of candidate interacting proteins of ZmPRN1. Table S3: Primers used in this study.
