# Supplementary material for: ZmPRN1 Negatively Regulates Salt Stress Tolerance by Modulating ROS Homeostasis in Maize (Zea mays L.)
Source: Plants (Basel). 2026 May 21;15(10):1585. doi: 10.3390/plants15101585 (PMC13210811; doi:10.3390/plants15101585)
Supplement: Supplementary file 1 [file plants-15-01585-s001.zip › plants-4309522-supplementary.pdf]

Supplementary Figures and Tables

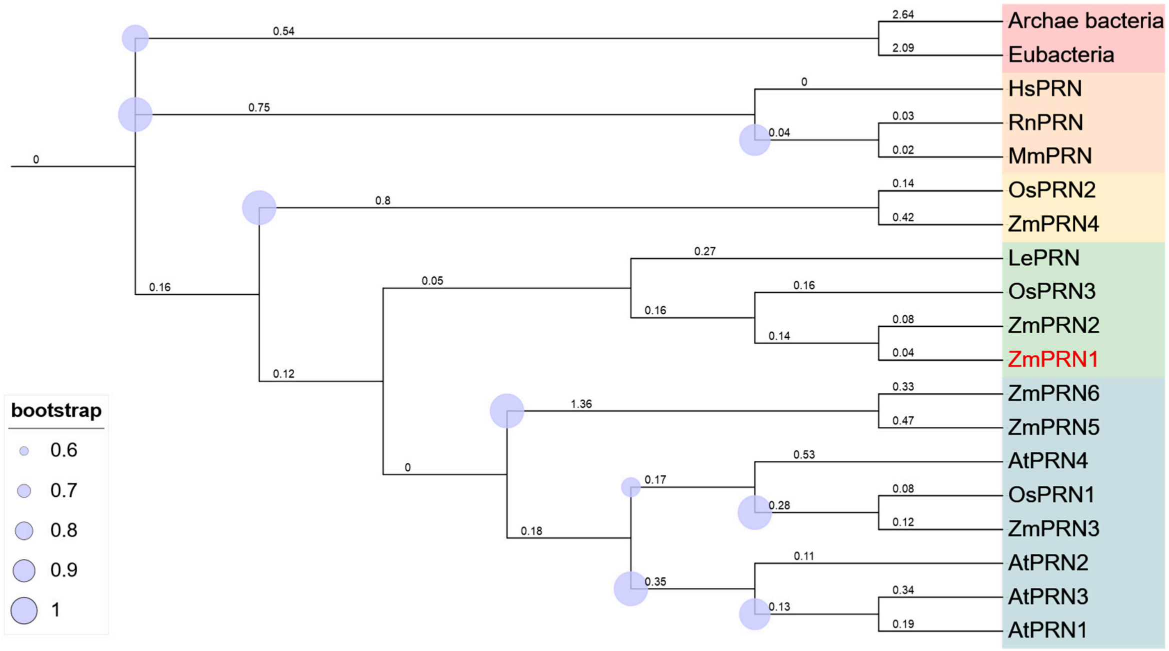

Figure S1. Phylogenetic tree of ZmPRN1 and its homologous proteins. The phylogenetic tree was constructed using the neighbor-joining (NJ) method. Bootstrap values (based on 1,000 replicates) are indicated at the nodes. The right panel lists the species or taxonomic groups of each protein, including Archaea, Eubacteria, and PRN homologs from various species (HsPRN from human, RnPRN from rat, MmPRN from mouse, OsPRN1-3 from rice, ZmPRN1-6 from maize, LePRN from tomato, and AtPRN1-4 from Arabidopsis). ZmPRN1 is highlighted with red font.

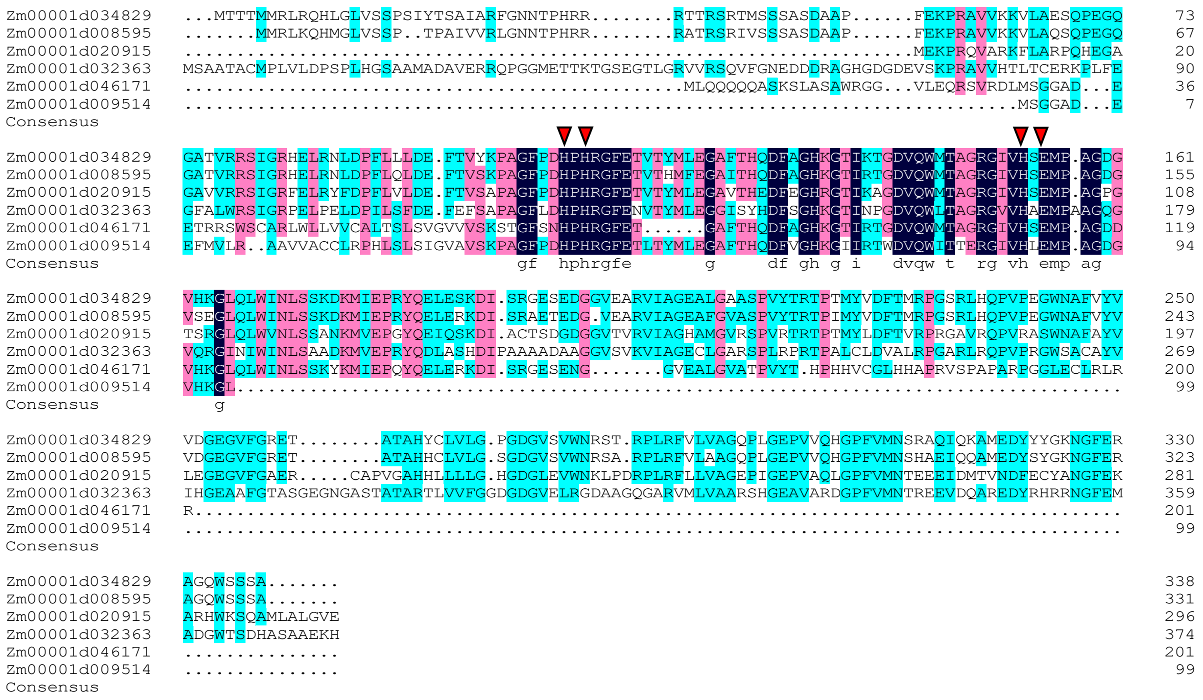

Figure S2. Amino acid sequence alignment of ZmPRNs. Multiple sequence alignment of PIRIN protein family members from maize. Conserved amino acid residues are shaded in dark, and similar residues are shaded in light. The N-terminal domain of PIRIN proteins is highly conserved. Four amino acid residues at the N-terminus, consisting of three histidine (His) residues and one glutamic acid (Glu) residue, are indicated by red arrows and form a conserved metal ion-binding site.

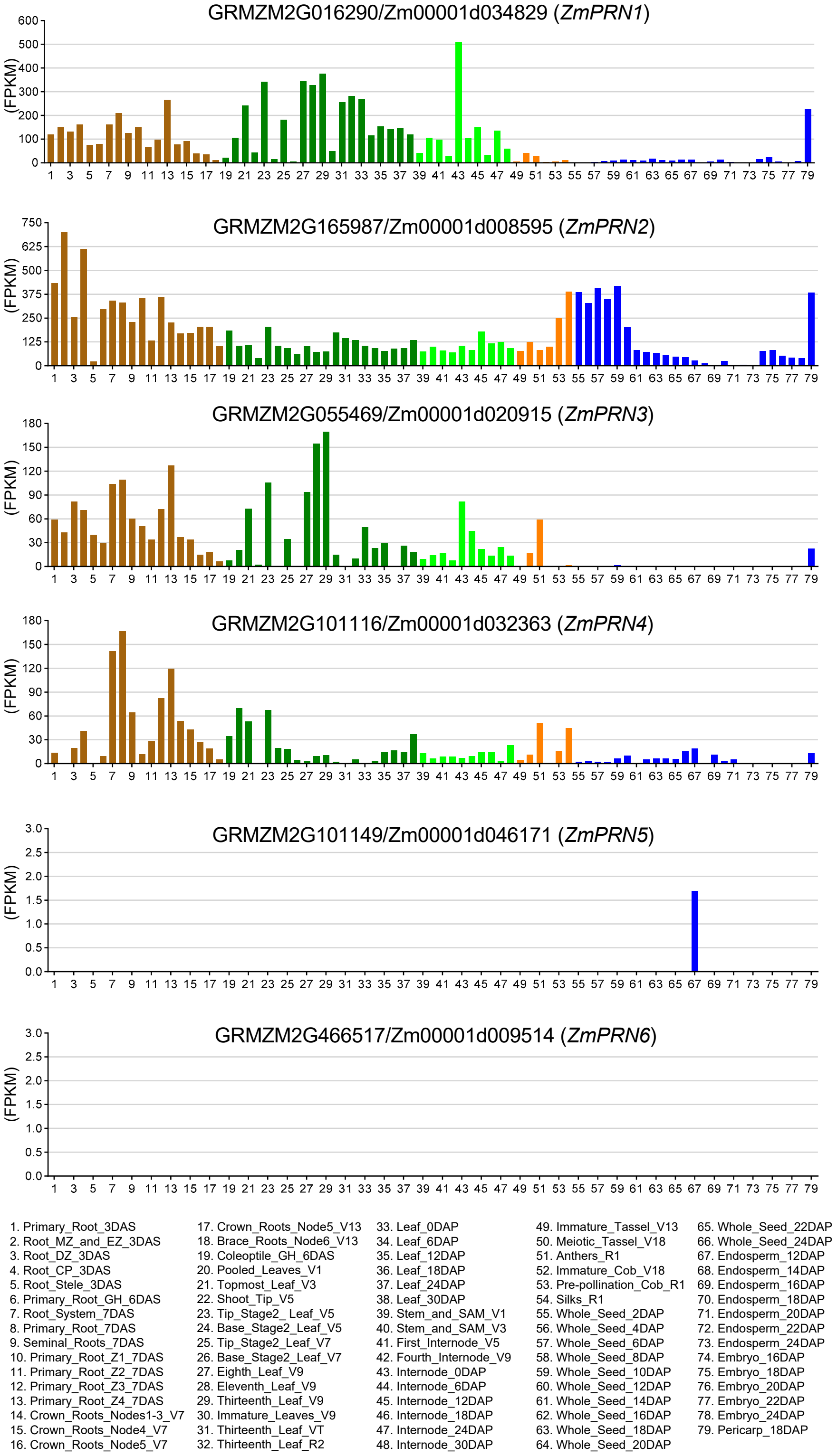

Figure S3. Expression atlas of six *ZmPRNs* in maize. Transcriptome data of maize throughout growth and development, including 79 different tissues/stages, obtained from MaizeGDB (www.maizegdb.org). FPKM, fragments per kilobase million.

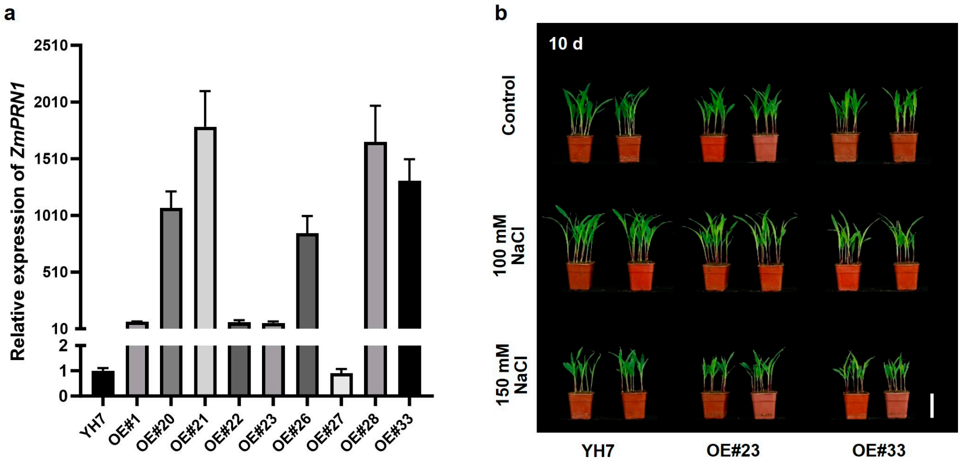

Figure S4. Phenotype of *ZmPRN1* overexpression lines treated with NaCl for 10 days. **(a)** Relative expression levles of *ZmPRN1* overexpression lines by RT-qPCR. **(b)** Comparision of wild-type and *ZmPRN1* overexpression lines treated with different concentration of NaCl for 10 days. Scale bar = 8 cm.

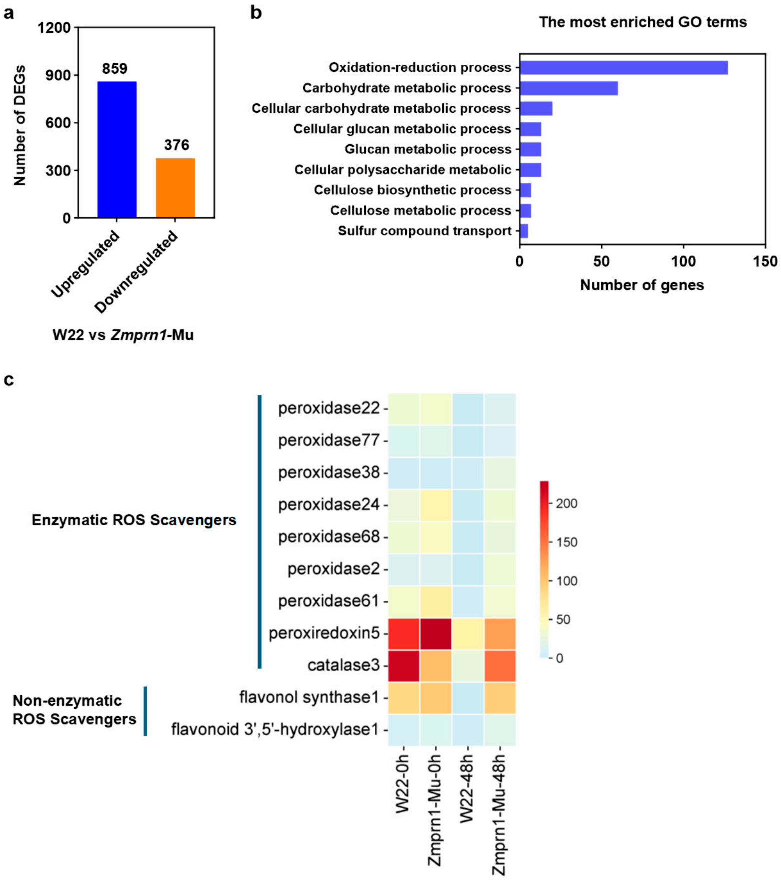

Figure S5. Transcriptomic profiling of the wild-type and *Zmprn1*-Mu seedlings under salt stress. **(a)** Numbers of differentially expressed genes between the wild-type and *Zmprn1*-Mu leaves after treatment with 200 mM NaCl for 48 h. A total of 859 up-regulated and 376 down-regulated genes were detected. **(b)** The top 9 enriched terms from the biological process category of Gene Ontology (GO) enrichment analysis are shown on the right, with "oxidation-reduction process" being the most significantly enriched term. (c) Heat map of enzymatic and non-enzymatic ROS scavenging genes in wild-type and Zmprn1-Mu leaves treated with 200 mM NaCl for 0 h and 48 h. The expression values (FPKM) are represented by the color scale.

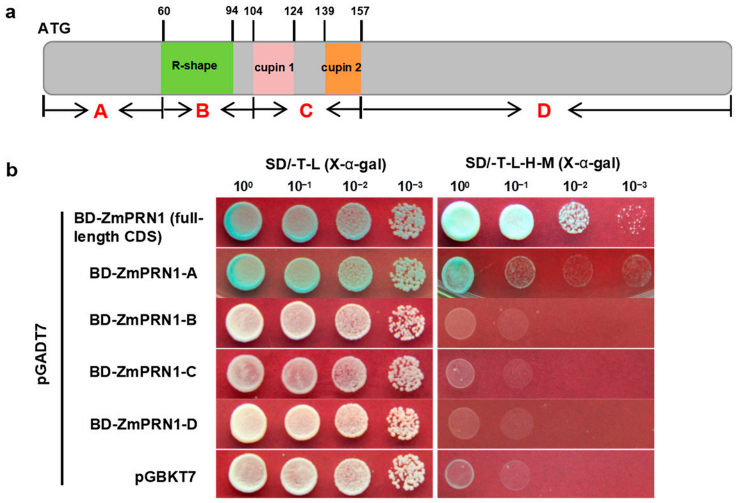

Figure S6. Functional domains and autoactivation activity of ZmPRN1. (**a**) Schematic representation of the functional domains of ZmPRN1. Based on the functional domain characteristics, the coding sequence (CDS) of ZmPRN1 is divided into four segments (A, B, C, and D). The positions of the R-shape domain and the conserved cupin 1 and cupin 2 domains are indicated. (**b**) Detection of autoactivation activity of full-length ZmPRN1 and its truncated segments. Full-length ZmPRN1 and segments A, B, C, and D were individually fused with the pGBKT7 vector and co-transformed with pGADT7 into the yeast strain AH109. Co-transformed yeast cells were serially diluted in 10-fold steps and spotted onto dropout medium (SD/-Leu/-Trp) and selective medium (SD/-Leu/-Trp/-His/-Ade) supplemented with X-α-gal. Autoactivation activity was assessed by yeast growth and blue color development on selective medium.

Table S1 Cis-acting elements in the promoter region of *ZmPRN1*

Function	Cis-acting element
Abscisic acid responsiveness	ABRE, ABRE3a, ABRE4
Salicylic acid responsiveness	TCA-element
Auxin responsiveness	AuxRR-core, TGA-element
MeJA-responsiveness	CGTCA-motif, TGACG-motif
Defense and stress responsiveness	TC-rich repeats

Table S2. Information of candidate interacting proteins of ZmPRN1

Sequence ID	The name of the protein
Zm00001d037473	WD40 -Transducin/WD40 repeat-like superfamily protein
Zm00001d045025	CPN10 - 20 kDa chaperonin chloroplastic
Zm00001d046449	ELFA9 - elongation factor 1-alpha
Zm00001d049641	GPC1 - Glyceraldehyde-3-phosphate dehydrogenase
Zm00001d010445	PYL9 - Abscisic acid receptor PYL9
Zm00001d011256	OHP3 - opaque2 heterodimerizing protein3
Zm00001d037774	IAA27 - Auxin-responsive protein IAA27
Zm00001d021515	NDF4 - NDH subunit F4
Zm00001d034998	RING371 - RING-type E3 ligase371
Zm00001d031168	GRP1 - glycine-rich protein1
Zm00001d016446	CCP1 - cysteine protease1

Table S3. Primers used in this study

Primer name	Sequence (5′-3′)	Application
ZmPRN1-Mu-F	AACAACACACCCCACCGCCGCCGAA	Mu insertion mutant genotyping
ZmPRN1-Mu-R	CACGCCGCCGTCCTCGCTCTCCCCG	Mu insertion mutant genotyping
TIR6	AGAGAAGCCAACGCCAWCGCCTCYATTTCGTC	Mu insertion mutant genotyping
qZmPRN1-F	GTATTTATACCTCCGCCATCGCC	RT-qPCR
qZmPRN1-R	AGGAGGAAGGGGTCCAGGTT	RT-qPCR
Tubulin 5-F	GCCGTTGCCGAGGTGTTC	Internal control (RT-qPCR)
Tubulin 5-R	GTCCTTCTCAAGAGCAGCCAAGT	Internal control (RT-qPCR)
